# A renal mycosis of roach (*Rutilus rutilus*) caused by the *Aureobasidium pullulans*

**DOI:** 10.1016/j.mmcr.2024.100652

**Published:** 2024-05-08

**Authors:** Jiří Řehulka, Vit Hubka

**Affiliations:** a Department of Zoology, Silesian Museum, Nadrazni okruh 31, 746 01, Opava, Czech Republic; b Department of Botany, Faculty of Science, Charles University, Benatska 2, 128 00, Prague 2, Czech Republic; c Laboratory of Fungal Genetics and Metabolism, Institute of Microbiology of the Academy of Sciences of the Czech Republic, v.v.i., Videnska 1083, 142 20, Prague 4, Czech Republic

**Keywords:** Black yeasts, Histopathology, Molecular diagnosis, Nuclear erythrocyte abnormalities, Spontaneous mycosis

## Abstract

Spontaneous mycosis caused by *Aureobasidium pullulans* is documented in roach in a cyprinid-prevalent water reservoir in Czechia. Gross pathological lesions included pale gills and splenomegaly, as revealed during necropsy examination. Histological examination showed extensive foci with fungal elements in the kidney. The isolated fungus was identified through phenotypic and molecular characterization, including phylogeny. This report represents the first case of *A. pullulans* infection in fish and cold-blooded vertebrates, to the best of our knowledge.

## 1 Introduction

*Aureobasidium pullulans* is a versatile melanized yeast-like fungus (*Ascomycota, Dothideomycetes, Dothideales*) well-documented for its wide distribution in different environments ranging from plant surfaces and soil to water and rock surfaces. It has been reported in cold and warm climates as well as humid and arid regions. Due to its wide spectrum of natural product synthesis, including enzymes and antimicrobial compounds, it is a significant candidate for biotechnological and environmental applications [1,2]. The fungus was described as a pathogen in a variety of human infections usually resulting from traumatic implantation, such as cutaneous and subcutaneous phaeohyphomycosis and keratitis. Additionally, it may cause fungemia or systemic infections affecting various organs in immunocompromised patients [3]. It is rarely reported in veterinary infections, limited to canine otitis [4], subcutaneous [5] and cerebral infections [6]. Cutaneous infections in porcupines (*Erethizon dorsatum*) have also been described [7]. Furthermore, the species was detected in a paraffin-embedded sample from feline nose tissue [8]. Along with *Rhodosporidium* sp., it was isolated from ocular infection in brown shrimp (*Penaeus californiensis*) [9].

The roach, *Rutilus rutilus*, is a non-predatory freshwater ray-finned fish, most commonly found in lowland aquatic ecosystems. As an eurytopic species, it is able to exploit all available habitats. The roach is one of the dominant species in the stable cyprinid phase of artificial lakes in Europe [10]. To the best of our knowledge, the present study is the first report describing mycosis due to *A. pullulans* not only in fish but also in cold-blooded vertebrates. The fungal pathogen was identified using phenotypic and molecular data.

## 2 Case presentation

**Fig. 1:**
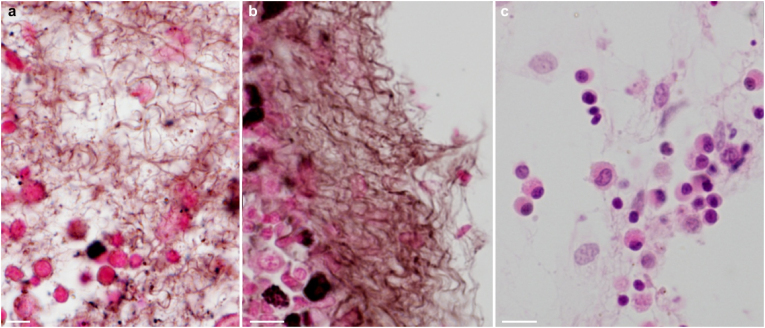
Mass of melanized fungal hyphae in kidney (a, b). Inflammatory cell population with abundant neutrophil band forms (c). Grocott's methenamine silver stain (a,b), May-Grünwald-Giemsa (c). Scale bars (a) = 5 μm, (b,c) = 10 μm.

During systematic health surveillance in fish stock of a water-supply reservoir 404 m above sea level in the Odra River basin, Czech Republic, mycosis was detected in a roach (*Rutilus rutilus*). The specimen, measuring 182 mm and weighing 140 g, exhibited pallor in the gills. Necropsy identified splenomegaly, and renal wet mounts revealed extensive fungal foci. GMS (Grocott's methenamine silver) stained kidney sections displayed infiltration by fungal hyphae (Fig. 1a and b) amidst cellular inflammation, characterized by a preponderance of neutrophil band forms (Fig. 1c). The fungus was absent in other organs examined (gill, liver, spleen, intestine, heart and brain), and no parasites or bacteria were found in the tissues analyzed.

**Fig. 2:**
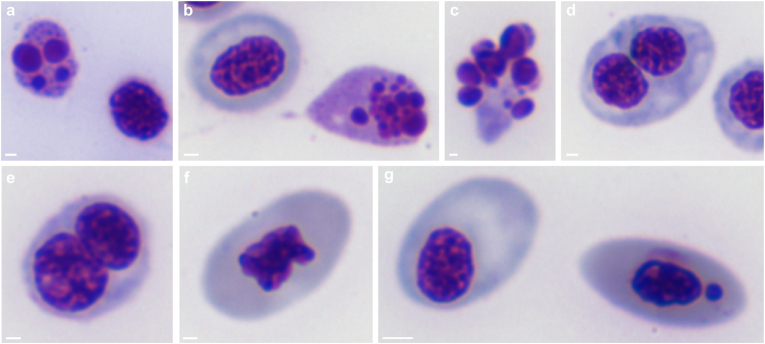
Nuclear abnormalities found during examination of peripheral blood cells. Erythrocytes with disintegrating nuclei (a–c). Binucleated cells (d, e). Lobed nuclei (f). Erythrocyte with eccentric nucleus (left) and micronucleated cell (g). May-Grünwald-Giemsa (a-g). Scale bars (a,b,d,e,f) = 1 μm, (c) = 0.5 μm, (g) = 2 μm.

Erythrogramme abnormalities were dominated by disintegrating nuclei, binucleated cells and eccentric nuclei (Fig. 2a–g). We used histological, bacteriological and mycological procedures described previously [11]. Haematological procedures used were described by Řehulka et al. [12].

A fungal strain was isolated in pure culture from the kidney on Sabouraud's agar at 24 °C. Bacteria were not observed in the lesion material and were not cultivated from samples inoculated on Blood agar (MKM 01011 Columbia agar; Trios), trypticase soya agar (MKM 10061; Trios), Mueller-Hinton agar (MKM 02011; Trios) and Anacker-Ordal agar. Parasitological investigation revealed only minor infestation by *Gyrodactylus prostae* (the intensity of infestation on fins and gills was two and three specimens).

**Fig. 3:**
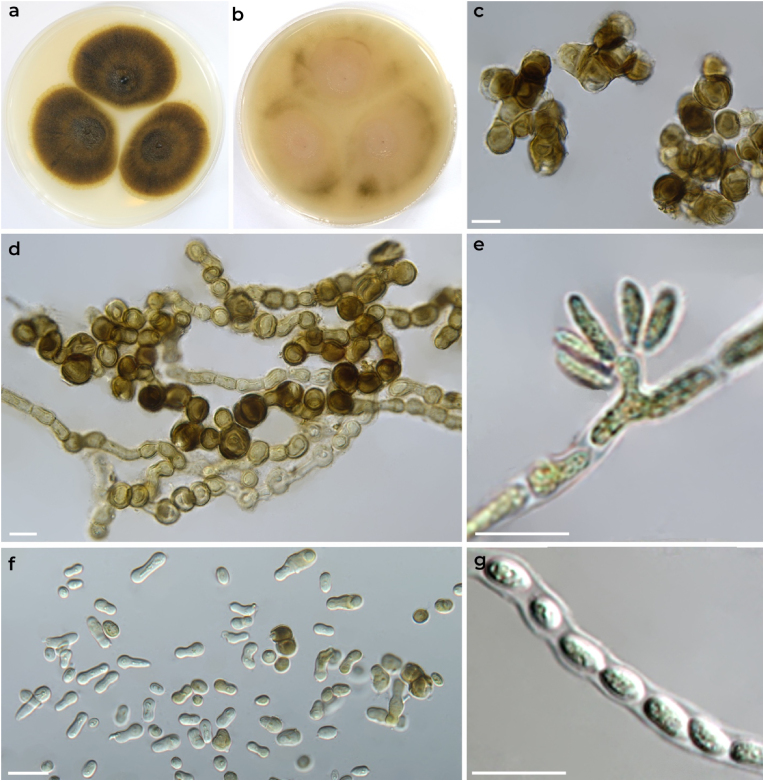
Macromorphology and micromorphology of *Aureobasidium pullulans* CCF 5287. Colonies incubated for 14 days on MEA (a) and PCA (b) at 25 °C. Clusters of chlamydospores (c); hyaline, pale yellow to brown vegetative hyphae with brown intercalar and terminal chlamydospores (d); conidiogenous cell integrated into vegetative hypha with hyaline conidia produced on a lateral protrusion (e); free yeast-like budding cells, conidia and chlamydospores (f); endoconidia (g). Scale bars: c-g = 10 μm. (For interpretation of the references to colour in this figure legend, the reader is referred to the Web version of this article.)

Subcultures of isolated strain were grown on malt extract agar (MEA, Oxoid), potato carrot agar (PCA, potatoes 20 g, carrot 20 g, agar 20 g, water 1000 ml) at 25 and 37 °C (Fig. 3). Colonies incubated at 25 °C on MEA reached a diameter of 23–26 mm after 7 days and 42–46 mm after 14 days. They were yellowish brown to brown, smooth, with a wet appearance and superficial exudate. Colonies on PCA reached a diameter of 32–38 mm after 7 days and 53–55 mm after 14 days. They were cream-coloured, pinkish-white with olive-brown sectors. No growth was observed at 37 °C.

Vegetative hyphae were 1.5–15 μm thick, consisting of hyaline thin-walled cells as well as yellowish to brown thick-walled cylindrical or subglobose cells that later disintegrated into 1- to 2-celled fragments (arthroconidia). Chlamydospores were mostly globose or subglobose, brown, thick-walled, intercalar or terminal, sometimes not clearly differentiated from thick-walled vegetative hyphae (continuum). Conidiogenous cells were integrated into vegetative hyphae, intercalar or terminal, with hyaline blastoconidia produced on small denticles and protrusions. Conidia were 1-celled, variable in shape (ellipsoidal, cylindrical, biconcave or pyriform), typically 5–19 × 3–7 μm in diameter. Yeast-like budding cells (conidia) were abundantly present, and occasionally endoconidia were found within vegetative hyphae (Fig. 3).

**Fig. 4:**
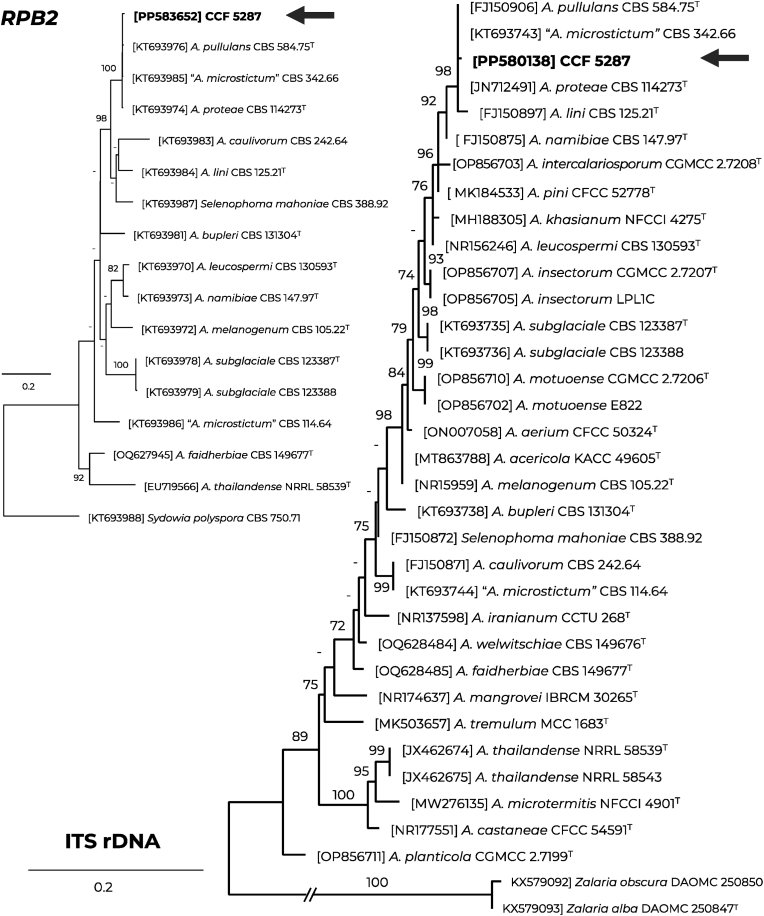
The best-scoring maximum-likelihood trees based on the ITS region (right) and the RNA polymerase II second largest subunit (*RPB2*) gene (left), illustrating the relationships of the case isolate of *Aureobasidium pullulans* CCF 5287 to species of *Aureobasidium*. The substitution models were selected and the tree was constructed using IQ-TREE v. 2.3.2 (ITS alignment: 35 taxa, 620 positions, 134, variable positions, TIM2e + I + G4 model; *RPB2* alignment: 17 taxa, 753 positions, 300 variable positions, TN + F + G4 model). Only bootstrap supports ≥70 % are shown, with lower supports indicated by a dash; the ex-type strains are designated by a superscript “T".

DNA isolation and PCR conditions used in this study were based on Hubka et al. [13]; the primers used for amplification of selected loci were based on Řehulka et al. [14]. A BLAST similarity search using the ITS region of rDNA revealed a 99.6 % similarity (2 bp difference) with *A. pullulans* ex-type CBS 584.75 (AJ244232) and its synonym *A. proteae*, while other species showed a similarity <99 %. Similarly, the *RPB2* gene (RNA polymerase II second largest subunit) showed the highest similarity with the ex-type of *A. pullulans* and *A. proteae* at 98.8 % (8 bp difference), while other species with available sequences in Genbank showed similarity <90 %. Phylogenetic trees constructed from ITS and *RPB2* loci confirmed the clustering of the case isolate with *A. pullulans* (Fig. 4). Other amplified markers were excluded from analyses due to low representation of sequences in GenBank (*tub2*; β-tubulin) or low variability between species (LSU rDNA). The sequences were deposited into the GenBank database under the following accession numbers: PP580138 (ITS), PP580189 (LSU), PP583653 (*tub2*) and PP583652 (*RPB2*). The fungus was deposited into the Culture Collection of Fungi, Charles University, Department of Botany, Prague, Czech Republic as CCF 5287.

## 3 Discussion

This case report documents the first known mycosis in *Rutilus rutilus* caused by the “black yeast” *A*. *pullulans*, a versatile environmental saprophyte and occasional pathogen with a significant presence in various aquatic ecosystems [15,16]. The observed pathology in roach primarily manifested as renal fungal foci, illustrating a novel aspect of *A. pullulans* pathogenic potential in poikilothermic animals. However, other clinical signs and hematological findings (disintegrating nuclei, binucleated cells and eccentric nuclei) cannot be explicitly attributed to fungal infection as no virological examination was performed. Such an examination could confirm the etiology of the morphological lesions of erythrocytes, which were similar to those seen in sockeye salmon infected with infectious hematopoietic necrosis virus (IHN) [17,18]. Binucleated cells have been previously described in young coho salmon [19] and young rainbow trout deficient in folic acid [20]. A tendency to produce binucleated cells was also observed in rainbow trout experimentally infected with the fungus *Bradymyces oncorhynchi* [12]. This finding prompts further exploration into the etiology of morphological lesions in erythrocytes and the potential role of the secondary metabolites produced by *A. pullulans* in terms of their toxicity, teratogenicity and mutagenicity. The described erythrocyte lesions, aside from micronucleated cells, were not observed in the other five examined roach from the same dam lake habitat.

The identification of *A. pullulans* in a wild freshwater fish expands its known opportunistic pathogenicity to cold-blooded vertebrates in natural settings, emphasizing the importance of comprehensive aquatic health monitoring. The specific pathogenic mechanisms of *A. pullulans* in *Rutilus rutilus* remain to be elucidated. While surface lesions on fish may serve as infection gateways, further research is needed to determine the exact dissemination routes, including potential transmission through contaminated food.

## CRediT authorship contribution statement

**Jiří Řehulka:** Conceptualization, Data curation, Formal analysis, Funding acquisition, Investigation, Methodology, Resources, Validation, Writing – original draft. **Vit Hubka:** Conceptualization, Data curation, Formal analysis, Investigation, Methodology, Resources, Software, Validation, Visualization, Writing – review & editing.

## Declaration of competing interest

None.
